# Supplementary material for: Global DNA Methylation and Hydroxymethylation Levels in PBMCs Are Altered in RRMS Patients Treated with IFN-β and GA—A Preliminary Study
Source: Int J Mol Sci. 2023 May 22;24(10):9074. doi: 10.3390/ijms24109074 (PMC10219581; doi:10.3390/ijms24109074)
Supplement: Supplementary file 1 [file ijms-24-09074-s001.zip › ijms-2335849-supplementary.pdf]

Supplementary information

## Table S1 – Significant correlation of epigenetic markers with clinical and demographic variables

| | | | | |
|---|---|---|---|---|
| a) | **Correlations of global levels of epigenetic markers with AGE** | | | |
| | **5mC** | ***r*** | ***p*** | ***n*** |
| | Total of participants | -0.339 | 0.0014 | 86 |
| | Healthy controls | -0.456 | 0.0130 | 29 |
| | RRMS patients | -0.265 | 0.047 | 57 |
| | Female participants | -0.359 | 0.009 | 52 |
| | Female controls | -0.507 | 0.027 | 19 |
| | Normal weight participants | -0.353 | 0.030 | 38 |
| | Normal weight controls | -0.552 | 0.033 | 15 |
| | Overweight female patients | -0.642 | 0.033 | 11 |
| | Participants ≤40 years old | -0.288 | 0.050 | 47 |
| | **AcH3** | ***r*** | ***p*** | ***n*** |
| | Total of participants | -0.223 | 0.039 | 86 |
| | Female participants | -0.316 | 0.021 | 53 |
| | Overweight female | -0.576 | 0.015 | 17 |
| | Obese female | -0.9 | 0.037 | 5 |
| | **AcH4** | ***r*** | ***p*** | ***n*** |
| | Total of participants | -0.236 | 0.028 | 87 |
| | RRMS patients | -0.308 | 0.020 | 57 |
| | Female participants | -0.34 | 0.012 | 54 |
| | Female participants | -0.411 | 0.017 | 33 |
| | Overweight female | -0.584 | 0.014 | 17 |
| | Treated patients | -0.286 | 0.042 | 51 |
| | IFN-β treated patients | -0.52 | 0.008 | 25 |
| b) | **Correlations of global levels of epigenetic markers with BMI** | | | |

| | | | | |
|---|---|---|---|---|
| | **5hmC** | ***r*** | ***p*** | ***n*** |
| | Healthy controls | 0.681 | < 0.0001 | 29 |
| | Female healthy controls | 0.585 | 0.011 | 18 |
| | Normal weight healthy controls | 0.581 | 0.029 | 14 |
| | Control ≤40 years old | 0.581 | 0.023 | 15 |
| | Control >40 years old | 0.694 | 0.012 | 12 |
| | GA treated patients | -0.45 | 0.027 | 24 |
| | **AcH4** | ***r*** | ***p*** | ***n*** |
| | Normal weight healthy controls | 0.786 | 0.021 | 8 |
| | **AcH3** | ***r*** | ***p*** | ***n*** |
| | Overweight healthy controls | 0.37 | 0.024 | 37 |
| c) | **Correlations of global levels of epigenetic markers with disease evolution** | | | |
| | **5mC** | ***r*** | ***p*** | ***n*** |
| | Obese patients | -0.755 | 0.03 | 8 |
| | <8 years of RRMS evolution | -0.494 | 0.008 | 28 |
| | >8 years of RRMS evolution | -0.439 | 0.017 | 29 |
| | **5hmC** | ***r*** | ***p*** | ***n*** |
| | <8 years of RRMS evolution | -0.379 | 0.047 | 28 |
| | <10 years of RRMS evolution | -0.42 | 0.015 | 33 |
| d) | **Correlations of global levels of epigenetic markers with time of DMT use** | | | |
| | **5mC** | ***r*** | ***p*** | ***n*** |
| | <8 years of RRMS evolution | -0.546 | 0.005 | 25 |
| | <10 years of RRMS evolution | -0.444 | 0.016 | 29 |
| | <5 years of DMT use | -0.627 | 0.003 | 20 |
| | **5hmC** | ***r*** | ***p*** | ***n*** |
| | Overweight patients | 0.45 | 0.047 | 20 |
| | ***AcH3*** | ***r*** | ***p*** | ***n*** |
| | <8 years of RRMS evolution | -0.416 | 0.039 | 25 |
| | <5 years of DMT use | -0.458 | 0.042 | 20 |

| | | | | |
|---|---|---|---|---|
| e) | **Correlations of global levels of epigenetic markers with EDSS** | | | |
| | **5mC** | ***r*** | ***p*** | ***n*** |
| | Male patients | -0.474 | 0.019 | 24 |
| | Overweight male patients | -0.626 | 0.039 | 11 |
| | **5hmC** | ***r*** | ***p*** | ***n*** |
| | Female patients | 0.431 | 0.017 | 30 |
| | Normal weight female patients | 0.585 | 0.036 | 13 |
| | **AcH4** | ***r*** | ***p*** | ***n*** |
| | Normal weight patients | -0.563 | 0.010 | 20 |
| | Normal weight female patients | -0.585 | 0.046 | 12 |
| f) | **Correlations of global levels of epigenetic markers with MSSS** | | | |
| | **5mC** | ***r*** | ***p*** | ***n*** |
| | Overweight male patients | -0.702 | 0.016 | 11 |
| | **5hmC** | ***r*** | ***p*** | ***n*** |
| | Female patients | 0.364 | 0.048 | 30 |
| | **AcH4** | ***r*** | ***p*** | ***n*** |
| | Normal weight patients | -0.513 | 0.021 | 20 |
| | Normal weight female patients | -0.629 | 0.028 | 12 |

## Table S2 – Summary of the correlations found in the subgroups of patients and controls

| | 5mC (%) | 5hmC (%) | AceH3 (O.D.) | AceH4 (O.D.) | BMI | Age (years) | EDSS | MSSS | EMRR evolution (years) | Time of DMT (years) |
|---|---|---|---|---|---|---|---|---|---|---|
| 5mC (%) | #subgroups<br>Direction<br>significance | | | | | | | | | |
| 5hmC (%) | **8<br>(+)<br>0.01 & 0.05** | #subgroups<br>Direction<br>significance | | | | | | | | |
| AceH3 (O.D.) | | | #subgroups<br>Direction<br>significance | | | | | | | |
| AceH4 (O.D.) | **2<br>(-)<br>0.05** | | | #subgroups<br>Direction<br>significance | | | | | | |
| BMI | | **6<br>(+, -)<br>0.0001–0.05** | **1<br>(+)<br>0.05** | **1<br>(+)<br>0.05** | #subgroups<br>Direction<br>significance | | | | | |
| Age (years) | **9<br>(-)<br>0.01 & 0.05** | | **4<br>(-)<br>0.05** | **7<br>(-)<br>0.01 & 0.05** | **3<br>(+)<br>0.05** | #subgroups<br>Direction<br>significance | | | | |
| EDSS | **2<br>(-)<br>0.05** | **2<br>(+)<br>0.05** | | **2<br>(-)<br>0.05** | **1<br>(-)<br>0.05** | **5<br>(+)<br>0.01 & 0.05** | #subgroups<br>Direction<br>significance | | | |
| MSSS | **1<br>(-)<br>0.05** | **1<br>(+)<br>0.05** | | **2<br>(-)<br>0.05** | **3<br>(-)<br>0.05** | **4<br>(+)<br>0.01 & 0.05** | **22<br>(+)<br>0.0001–0.01** | #subgroups<br>Direction<br>significance | | |
| EMRR evolution (years) | **2<br>(-)<br>0.01 & 0.05** | **2<br>(-)<br>0.05** | | | | **3<br>(+)<br>0.01 & 0.05** | | **5<br>(+, -)<br>0.01 & 0.05** | #subgroups<br>Direction<br>significance | |
| Time of DMT (years) | **4<br>(-)<br>0.01 & 0.05** | **1<br>(+)<br>0.05** | **2<br>(-)<br>0.05** | | | **1<br>(+)<br>0.05** | | **6<br>(-)<br>0.01** | **20<br>(+)<br>0.0001–0.01** | #subgroups<br>Direction<br>significance |

*#subgroups, number of subgroups with that specific significant correlation; Direction, positive (+) or negative (-) correlation; significance, significant p values found across de subgorups.*

## Table S3 – Comparison of epigenetic marker levels in patients with RRMS and controls, stratified by sex.

| Var. | Subgroup | | n | M±SD | *p* |
|---|---|---|---|---|---|
| **%5mC** | RRMS Patient | Female | 33 | 14.01 ± 5.6 | 0.102[a] |
| | | Male | 24 | 15.5 ± 7.56 | |
| | Controls | Female | 19 | 20.04 ± 11.16 | |
| | | Male | 10 | 20.46 ± 7.73 | |
| **Var.** | **Subgroup** | | **n** | **M±SD** | ***p*** |
| **%5hmC** | RRMS Patients | Female | 32 | 0.0017 ± 0.0008 | 0.018*[a] |
| | | Male | 24 | **0.0014 ± .0007†** | |
| | Controls | Female | 20 | 0.0015 ± .0012 | |
| | | Male | 10 | **0.0024 ± 0.0007†** | |
| **Var.** | **Subgroup** | | **n** | **M±SD** | ***P*** |
| **AcH3 (O.D.)** | RRMS Patients | Female | 33 | 0.1145 ± .039 | 0.065[a] |
| | | Male | 24 | 0.11 ± 0.032 | |
| | Controls | Female | 20 | 0.1217 ± 0.043 | |
| | | Male | 9 | 0.1097 ± 0.069 | |
| **Var.** | **Subgroup** | | **N** | **M±SD** | ***P*** |
| **AcH4 (O.D.)** | RRMS Patients | Female | 36 | 0.101 ± 0.02 | 0.189[a] |
| | | Male | 24 | 0.1024 ± 0.02 | |
| | Controls | Female | 20 | 0.0951 ± 0.013 | |
| | | Male | 9 | 0.1027 ± 0.01 | |

*Data are expressed as M ± SD. (*[a]p ≤ 0.05, Kruskal-Wallis test; †p <0.01, Dunn's test). RRMS, relapsing-remitting multiple sclerosis. %5mC, percentage of 5-methylcytosine; %5hmC, percentage of 5-hydroxymethylcytosine; O.D., optical density; AcH3, Histone H3 acetylation; AcH4, Histones H4 acetylation.*
